# Large Hepatic Subcapsular Hematoma Following Endoscopic Retrograde Cholangiopancreatography: A Case Report

**DOI:** 10.7759/cureus.21920

**Published:** 2022-02-05

**Authors:** Rahul Arya, Rajeev N Priyadarshi, Tanmoy Maji, Ramesh Kumar, Utpal Anand

**Affiliations:** 1 Gastroenterology, All India Institute of Medical Sciences, Patna, IND; 2 Radiology, All India Institute of Medical Sciences, Patna, IND; 3 Surgical Gastroenterology, All India Institute of Medical Sciences, Patna, IND

**Keywords:** endoscopic retrograde cholangiopancreatography, abdominal pain, hepatic subcapsular hematoma, angioembolization, imaging, ercp complications

## Abstract

For decades, endoscopic retrograde cholangiopancreatography (ERCP) has been the cornerstone in the treatment of several biliopancreatic diseases. Although it is a relatively safe procedure, there are certain hazards involved. Hepatic subcapsular hematoma (HSH) is an uncommon complication of ERCP, with only a few cases reported in the literature to date. We present here a case of large HSH that developed 48 hours after an otherwise uneventful ERCP for choledocholithiasis. After being apparently well for the first two days post-ERCP, the patient began to develop abdominal pain and restlessness associated with hemodynamic instability and a decline in hemoglobin levels. Computed tomography (CT) confirmed the presence of a large HSH. The patient was managed nonsurgically with vascular angioembolization followed by ultrasound-guided percutaneous catheter drainage of hematoma. This case highlights the necessity of increasing awareness about this complication in order to aid in early diagnosis and management.

## Introduction

Endoscopic retrograde cholangiopancreatography (ERCP) is a commonly used procedure that has revolutionized the management of biliopancreatic diseases. Compared to surgical alternatives, ERCP has the advantage of being a less-invasive and cost-effective procedure. However, because ERCP is a high-risk endoscopic procedure associated with various complications, its primary role has switched from diagnostic to therapeutic [1]. According to a recent multicentric database registry for ERCP in Japan, the overall complication rate of ERCP was 10.6%, with a 30-day mortality rate of 1.1% [2]. The common complications of ERCP include acute pancreatitis, post sphincterotomy bleeding, duodenal perforation, and infection (cholangitis) [3]. Post-ERCP HSH is an extremely rare complication that was first described in the year 2000 and has only a few case reports in the literature [4]. We present a case of a large HSH that was diagnosed 48 hours after an ERCP and was effectively treated with angioembolization and percutaneous drainage.

## Case presentation

A 60-year-old female with a history of hypertension and hypothyroidism who was on regular medications presented to us with upper abdominal pain and mild jaundice for 15 days. At the time of presentation, she was icteric, afebrile, and hemodynamically stable, and her abdominal examination was unremarkable. Her liver function tests (LFTs) revealed a cholestatic pattern of liver enzymes with elevated serum bilirubin and alkaline phosphatase, while her abdominal ultrasonography (USG) showed multiple calculi in the gall bladder with choledocholithiasis causing biliary obstruction.

**Figure 1:**
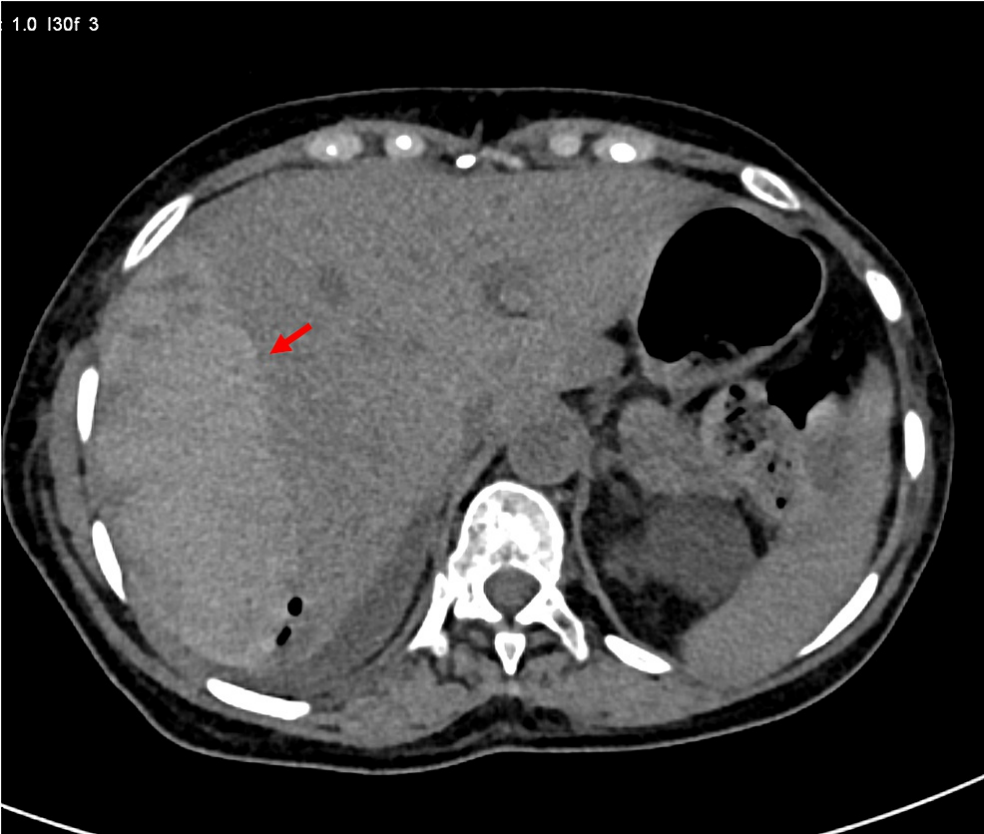
Axial non-contrast CT image showing the hyperdense subcapsular fluid collection indicating hematoma (arrow)

**Figure 2:**
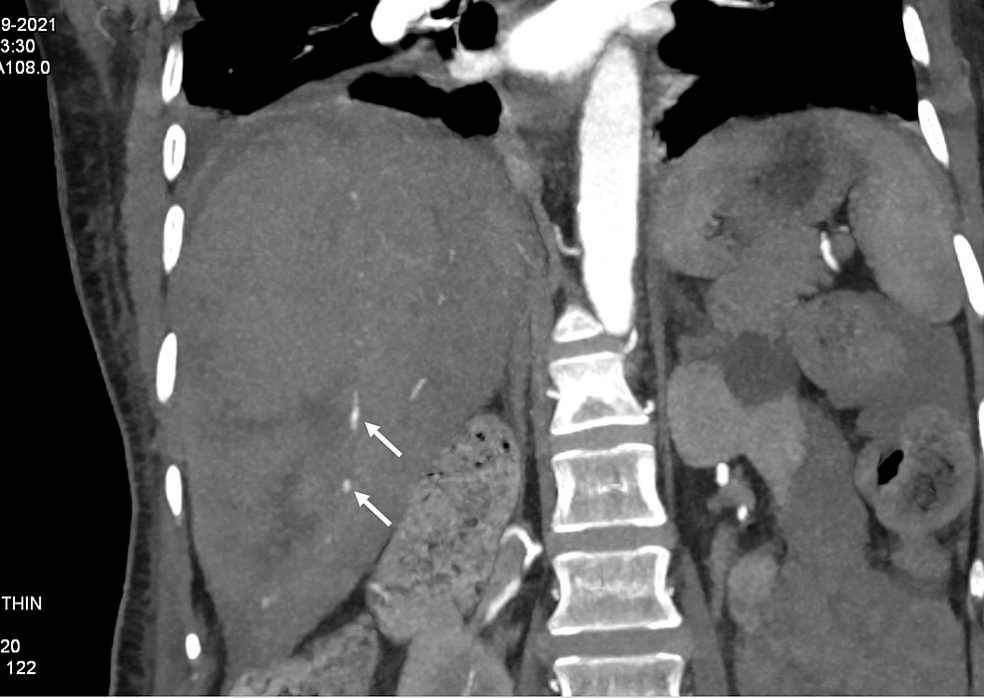
CT angiography showing the nodular extravasation of contrast outlining the hematoma (arrows)

ERCP was performed for choledocholithiasis. A 0.025-inch, straight, hydrophilic tip guidewire (VisiGlide Guidewire, Olympus, Westborough, MA, USA) was used to selectively cannulate the common bile duct (CBD). Her cholangiogram revealed an 11-mm dilated CBD with a 9-mm round stone at the lower end. The stone was easily swept out with the use of an extractor balloon following biliary sphincterotomy. The patient remained apparently well following ERCP and was under observation in the hospital for monitoring of liver enzymes. However, on day three, when she was scheduled for discharge from the hospital, she developed right upper abdominal pain along with restlessness. On examination, she was pale, and she had tachycardia with hypotension. An immediate blood investigation revealed a significant drop in hemoglobin from 10.1 g/dL (pre-ERCP) to 4.1 g/dL. On screening USG, a large hematoma was detected in the subcapsular region of the right hepatic lobe. Following that, a computed tomography (CT) scan of the abdomen was performed with CT angiography, which revealed a large heterogeneous lesion (12.3 × 5.2 × 13.7 cm) with air foci within the subcapsular region of the right hepatic lobe, indicating a large HSH (Figure 1). The hematoma was outlined by nodular extravasation of contrast on CT angiography (Figure 2).

**Figure 3:**
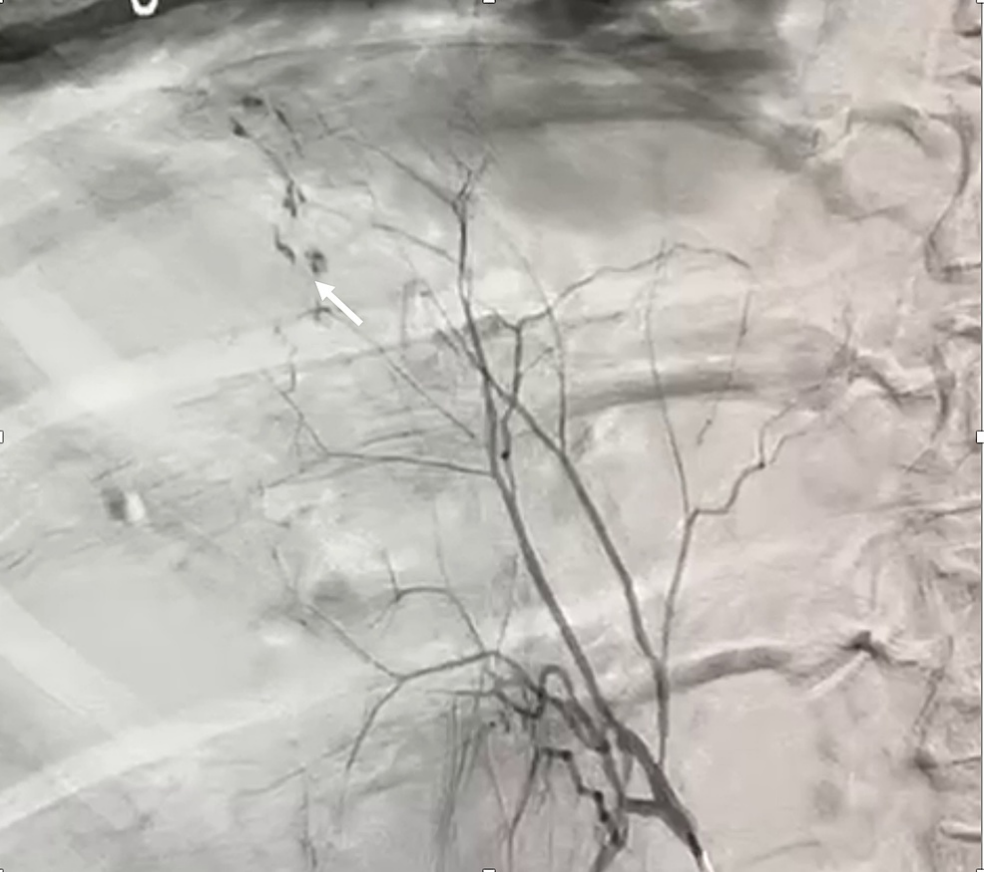
Selective catheter angiography of the right hepatic artery shows extravasation (arrow) of contrast from subcapsular arteries

**Figure 4:**
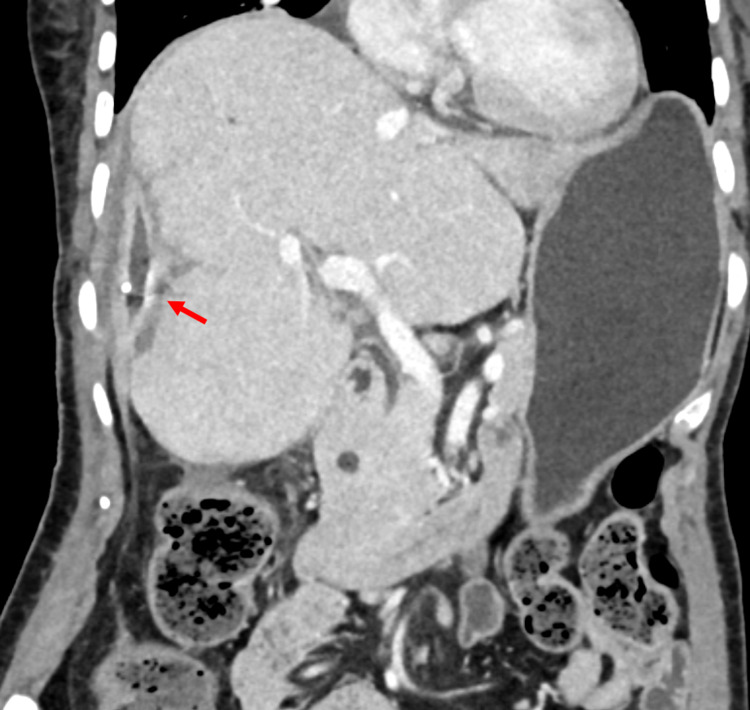
Contrast-enhanced CT performed one month after the ERCP and 20 days after catheter drainage showing almost complete disappearance of perihepatic fluid collection (arrow)

Catheter angiography of the celiac axis was then performed under local anesthesia, which showed ectatic vessels at the hepatic surface (Figure 3). Selective catheterization of the right hepatic artery was performed, and ectatic vessels were embolized using polyvinyl alcohol (PVA) particles. A repeat angiogram confirmed the devascularization of ectatic vessels. The patient was managed with packed red cell transfusions, antibiotics, and other supportive care. Although her hemoglobin levels remained rather stable, she developed fever along with a cough. Her CT thorax revealed consolidation involving the right lower lobe, suggesting lobar pneumonia, and the lower cuts of her chest CT revealed further expansion of hematoma. The hematoma was planned to be drained, and percutaneous catheter drainage (PCD) was chosen in view of her overall health condition. With continued PCD and upgradation of antibiotics, her pneumonia subsided, and her general condition improved remarkably. A CT scan done six weeks later showed near-complete resolution of HSH (Figure 4).

## Discussion

HSH is an extremely rare complication of ERCP that, if not recognized and treated promptly, can be fatal. Ortega et al. were the first to describe it in the year 2000 [5]. In the literature, 61 cases of post-ERCP HSH have been reported to date, with an overall mortality rate of 7.5% [4]. This may be an underdiagnosed condition because routine imaging is not usually performed after ERCP, and this explains the low incidence of this complication [6].

The pathophysiological mechanism of such hematomas is not fully understood, and several hypotheses have been proposed. One theory proposes that during biliary clearance using a balloon, tractional pressures develop, causing small biliary blood vessels to rupture, resulting in intraparenchymal hemorrhage and subcapsular collection due to centrifugal flow of blood [5]. According to another theory, the guidewire used in ERCP might cause injury to small caliber vessels in the biliary tree, resulting in injury and hemorrhage [7]. This mechanism would also justify the presence of air in the hematoma. However, HSH has also been reported when CBD stone was extracted without using a guidewire. Moreover, there are also few published reports of unexplained HSH following laparoscopic cholecystectomy [8]. The anatomic and hemodynamic characteristics of this region, such as negative subdiaphragmatic pressure and a “third inflow” of blood from other sources, may also play a role [9]. Finally, the presence of abnormal ectatic vessels on the liver surface, as found in our case, might indicate the role of some vascular malformations.

HSH can be suspected when, after ERCP, patients develop sudden-onset upper abdominal pain associated with tachycardia and hypotension. HSH can take up to 10 days to appear after an ERCP procedure [10]. In our case, it was diagnosed on day 3. Diagnosis is confirmed by imaging modalities such as USG and CT scan [11]. The outcome in these cases in general is good, provided it is diagnosed early and treated accordingly. The treatment of HSH should be individualized for each patient. Patients with a small confined hematoma who are hemodynamically stable can be treated conservatively with blood transfusions, intravenous fluids, prophylactic antibiotics, and analgesics. When a patient has severe anemia and an expanding hematoma, an immediate angiography with angioembolization of bleeding vessels, if detected, should be considered. Surgical treatment should be reserved for patients with ruptured HSH with intraperitoneal collections and signs of peritonitis [12].

## Conclusions

HSH is a rare but dreadful complication of ERCP. It should be suspected in patients who develop clinical features such as abdominal pain, pallor, and hypotension after ERCP, along with laboratory features showing reduced hemoglobin and fall in hematocrit levels. Although the exact cause of this complication is unknown and mortality has been described in a few cases, a prompt diagnosis with cross-sectional imaging can lead to a favorable outcome in most patients following conservative management. Rapidly expanding hematomas and patients having hemodynamic instability require urgent selective catheter angioembolization of bleeding vessels. Surgery is warranted in cases of ruptured hematomas with signs of peritoneal inflammation.
